# A first voice perspective of people experiencing homelessness on preferences for the end-of-life and end-of-life care during the COVID-19 pandemic

**DOI:** 10.1186/s13104-022-06025-z

**Published:** 2022-04-15

**Authors:** Cait Vihvelin, Viraji Rupasinghe, Jean Hughes, Jeff Karabanow, Lori E. Weeks

**Affiliations:** 1 School of Nursing, Dalhousie University, Halifax, NS B3H 4R2 Canada; 2 School of Social Work, Dalhousie University, Halifax, NS B3H 4R2 Canada

**Keywords:** Homelessness, Palliative, End-of-life, Death, Dying, COVID-19

## Abstract

**Objective:**

People experiencing homelessness often encounter progressive illness(es) earlier and are at increased risk of mortality compared to the housed population. There are limited resources available to serve this population at the end-of-life (EOL). The purpose of this study was to gain insight into preferences for the EOL and end-of-life care for people experiencing homelessness. Utilizing an interpretive phenomenology methodology and the theoretical lens of critical social theory, we present results from 3 participants interviewed from August to October 2020, with current or previous experience of homelessness and a diagnosis of advanced disease/progressive life-threatening illness.

**Results:**

A key finding focused on the existential struggle experienced by the participants in that they did not care if they lived or died. The participants described dying alone as a bad or undignified way to die and instead valued an EOL experience that was without suffering, surrounded by those who love them, and in a familiar place, wherever that may be. This study serves to highlight the need for improvements to meet the health care and social justice needs of people experiencing homelessness by ensuring equitable, humanistic health and end-of-life care, particularly during the context of the COVID-19 pandemic.

**Supplementary Information:**

The online version contains supplementary material available at 10.1186/s13104-022-06025-z.

## Introduction

People experiencing homelessness are at an increased risk of mortality, with the mean age of death being 34–47 years in Canada [1–3]. Eight out of 10 people who experience homelessness also experience chronic and/or progressive health conditions including chronic obstructive pulmonary disease, diabetes, tuberculosis, cancer, and HIV/AIDS [3]. Unintentional injuries, hypothermia, heatstroke, alcohol, and drug overdose are also prevalent among this population [3, 4].

People experiencing homelessness are profoundly stigmatized which furthers the adverse health outcomes and unmet needs for this population, including difficulties in accessing healthcare and receiving a lower standard of care than the general population [5, 6]. People experiencing homelessness along with concurrent disorders, such as mental health issues and substance use disorders, are particularly disadvantaged as they face barriers to healthcare services, high rates of hospitalization and discrimination from healthcare providers [7, 8].

Access to high quality end-of-life care (EOLC) is a basic human right [9]. Although relatively little research has been done regarding EOLC services for people experiencing homelessness, there is high demand for EOLC for this population [2, 4, 10, 11]. Yet, EOLC services are underused among this population [12, 13] due to inexperience or lack of education for healthcare workers, stigma within the healthcare system, and mistrust between healthcare professionals and people experiencing homelessness [1, 2, 13–15]. In many institutions, zero tolerance policies exist, prohibiting the use of alcohol and other substances which makes care inaccessible for many people [16, 17]. Moreover, there are limited resources available that specifically serve the multifaceted EOLC needs for this population [9, 15–17]. It is not surprising that people experiencing homelessness avoid EOLC [17].

While there is a growing body of knowledge on this topic, few studies have been conducted from the perspective of people experiencing homelessness and living with a progressive life limiting illness and many studies rely on evidence from health and social service providers. In one relevant systematic review, only 4 of 9 included studies included any data collected from people experiencing homelessness [14]. The purpose of this study was to gain insight into preferences for the EOL and EOLC from people experiencing homelessness.

## Main text

### Methods

#### Data collection

Semi-structured telephone interviews [18] of approximately one hour were audio recorded by the first author who then created a verbatim transcript enhanced with notes to document behaviors such as silence and laughter. The interview guide was developed for this study (see Additional file 1).

#### Setting and recruitment

After receiving research ethics board approval, the first author contacted services in the Halifax Regional Municipality in Nova Scotia, Canada, by telephone or email that served people experiencing homelessness (e.g., a street navigator). For those who provided authorization, the first author sent a recruitment poster including study details and contact information. Potential participants contacted the first author via telephone, and they were screened for the following eligibility criteria: ability to speak and understand English; over the age of 18; access to a phone; current or previous experience of homelessness; a diagnosis of advanced disease/progressive life-threatening illness (e.g., COPD, cancer, HIV/AIDS, substance addiction); and the cognitive capacity to consent to participating in the study. Once eligibility was established and any questions about the study were answered, informed consent was obtained. Participants received a $25 honorarium.

Interviews with three people were conduced from August-October 2020, which was reasonable given recruitment challenges during the COVID-19 pandemic. Having a small sample size is an advantage to understand the richness and depth of lived experiences.

#### Data analysis

We used an inductive thematic analysis approach [19] involving reflective writing and the stages of identifying an early focus and lines of inquiry, central concerns, exemplars and paradigm cases, shared meaning, and final interpretations. [19]. Pseudonyms protected participant’s privacy and humanized their stories.

Research rigor was ensured in various ways. Draft themes were identified by the first author and discussed with the last author throughout the data analysis process. While space is limited in this report, we provided descriptions and quotes which supports the transferability of the findings [20]. A reflexive journal was kept throughout the entire study to examine personal awareness, assumptions, and biases and to create a level of transparency [21].

### Results

#### The participants

The participants included two males and one female whose ages ranged from 36 to 60.

“Nate” lived in an assisted living environment for those with chronic alcohol dependency; previously he stayed at various shelters. During the COVID-19 pandemic he was sheltered in a gymnasium and then provided with a hotel room by a local non-profit organization. “Lilli” and “Darcy” were involved romantically, and they were living in a tent prior to being provided a hotel room for a few weeks. They were unsure of their living situation following the interview.

Nate had many health conditions including chronic alcoholism, high blood pressure, and mental health issues. Lilli was a former Licenced Practical Nurse was diagnosed with Stage 3 stomach cancer, depression, bipolar disorder, borderline personality disorder, and she struggled with addictions. Lilli had explored medically assisted dying. Darcy struggled with post-traumatic stress disorder, anxiety, addictions, and untreated seizures.

#### Existential struggle

Each participant experienced an existential struggle related to death. Nate voiced that he did not care if he lived or died because he felt that he had nothing meaningful left. “I don’t really care if I live or die; it’s all gone, there’s nothing left of me. So I don’t really care about life or death. I don’t care if I get Corona. There’s nothing left for me. My life is all gone. My good life is all gone.” Lilli thought about dying when she experienced pain and during the time of her stomach cancer diagnosis; she didn`t want to get sicker but also didn`t care if she got better: “About passing away? Sometimes if I am having a shitty day, if I’m having a bad day ya know, it makes me think if I have to struggle like this so bad and be in so much pain what is the point of still carrying on.” Darcy`s voiced that dying in a violent manner or dying in general is something that he thought about “all the time.”

#### I don’t want to suffer

The participants described suffering to be the epitome of what constitutes a “bad” death, such as suffering related to a cancer diagnosis, dying by suicide, or in a tragic or violent way. In the past, the participants encountered traumatizing acts of violence and have lost acquaintances through the act of suicide which impacted their perceptions of undignified deaths. Darcy voiced that “A bad death would be getting hurt or murdered or anything like that or getting hit by a car. Ya know that would be a bad death to me.” The participants also voiced their ideal EOL situation would involve just going to sleep and not waking up or to grow old and die without suffering.

#### I want to die at home, wherever that may be

This theme explores the uniqueness of dying at home as a desire for those whom others may view as being ‘without’ a home. Home may be a shelter, tent or park bench, but the act *of dying at home* remains a relevant desire and preference. The support needed to die comfortably at home can be intensive and requires access to many services such as palliative and home care, which can create a barrier for dying at home for people experiencing homelessness. For example, Lilli explained: “Yea I would want to be at home, I wouldn’t want to be in assisted living or a hospital or anything like that, I’d rather just be at home; Yea I don’t want to go in like palliative care and have people that I don’t know looking after me.’

#### Surrounded by those who love me

The participants voiced the importance of being surrounded by loved ones. Lilli explained that “a bad death would be passing away by myself and not having my family with me.” Darcy voiced how he had lost ties with his family, but he has been encouraged by Lilli to reunite with them. Despite the potential to reunite with family, Darcy voiced that he would not want them present in an EOL situation. He said: “No I`d just want her.”

#### Dying without ‘nothing’

Dying without ‘nothing’ was identified as a particular concern by Nate. He indicated how he perceived his current quality of life as being poor. Feeling like there was nothing of value left in his life, whether that be his health or financial means was a central concern regarding his attitude and beliefs surrounding EOL. “That’s a bad death, dying asleep and not waking up, dying without nothing.”

### Discussion

This results of this study are important in that it is among one of a small number to explore preferences for EOL and EOLC from the perspective of people experiencing homelessness and living with progressive illness(es) [10, 16, 17]. In addition, as the interviews were conducted during the COVID-19 pandemic, the results provide insights into the unique challenges experienced by people experiencing homelessness during this difficult time.

A key finding focused on existential struggle experienced by the participants in that they did not care if they lived or died. Their struggle was related to their thoughts about dying and the dying process, as well as the perceived lack of meaning or purpose in their lives. Little is known about the existential struggle of people experiencing homelessness, and especially among those in EOL situations [22]. Our results indicate that there is need to explore how to provide person-centered care including mental, spiritual, and psychosocial care for people living with progressive illness(es) in an EOL context. While there are established standards developed for training for professionals working in palliative care on specific topics, such as spiritual care [23], this training should also encompass the needs of people experiencing homelessness. In addition, efforts are needed to help people experiencing homelessness to have confidence in health care providers and be treated with equality and respect [24].

It was evident that suffering, dying alone and ‘without nothing’ were central concerns. These concerns are heightened for people experiencing homelessness. The participants identified a “good” death, or an ideal EOL situation, would be dying in one’s sleep, without pain or suffering and surrounded by those who loved them. These results are supported by the small body of literature available that looks at important aspects of EOLC for those experiencing homelessness. According to the literature, the discourse that has been identified surrounding what constitutes a “good” death, or ideal EOL situation for this population include: the desire for a peaceful death that is without pain or suffering, making a spiritual or religious connection, making amends with family and friends, and seeking reconciliation or taking care of inner conflicts [25–27].

Our results also highlight the importance of broadening the definition of family as EOL caregiver. For some people experiencing homelessness, “family” may be non-traditional (e.g., street family, shelter workers, street navigators). Stajduhar and colleagues recommend challenging the assumptions and policies that prevent non-traditional family from caring for their loved ones at the EOL [11]. In addition, for people experiencing homelessness, the provision of care at the EOL at home may include a shelter, tent, or park bench. It is clear that the limited resources are not adequate to meet the needs of people experiencing homelessness a the EOL [10, 16, 17].

The findings have the potential to inform the education and practice of healthcare providers in various settings. However, to implement findings into upstream health systems planning, there is a need for additional research that includes feasibility data, such as large scale qualitative or quantitative studies, that can prove the need for specialized homelessness EOLC services at a local level. The voice of people experiencing homelessness should be at the forefront of any new initiatives to ensure plans are effective and serve to strengthen outreach and engagement efforts [28].

### Limitations

Recruitment during the COVID-19 pandemic was particularly challenging. The small sample size limits transferability of the findings [13].Two of the participants in this study were involved in a romantic relationship and living together at the time of interviewing.

## Supplementary Information

**Additional file 1. **Semi-structured interview guide.

## Data Availability

The data analysed during this study are not publicly available as the qualitative data may be identifiable. Questions about access to deidentified data should be addressed to the corresponding author.

## Abbreviations

EOL: End-of-life
EOLC: End-of-life care
