# Supplementary material for: A first voice perspective of people experiencing homelessness on preferences for the end-of-life and end-of-life care during the COVID-19 pandemic
Source: BMC Res Notes. 2022 Apr 15;15:142. doi: 10.1186/s13104-022-06025-z (PMC9012062; doi:10.1186/s13104-022-06025-z)
Supplement: Supplementary file 1 — Additional file 1. Semi-structured interview guide. [file 13104_2022_6025_MOESM1_ESM.docx]

**Additional file 1. Semi-structured interview guide**

1. When you saw my poster, what was it that made you interested in speaking with me?
2. I’d like to start off by learning about you and your history. Tell me a bit about yourself (probes: age, housing history, employment history, self-identified gender).
3. Tell me about your health and any life-limiting illnesses you may have? What has it been like recently?
4. Tell me more about your healthcare experience since you have been experiencing homelessness. Try describing a specific example of a time when you felt really unwell and what happened.
5. What does (or would) end-of-life care look like for you? (probes: Tell me about any concerns you might have with end-of-life care, what would an ideal end-of-life care situation look like for you?)
6. What would a “good death” look like for you? What would a “bad death” look like for you?
7. Based on what you know about end-of-life care/palliative care, what would you like to see be done differently?
